# Asphyxia Neophytorum, an Improved Method of Treatment*Read before the Medical Association of Georgia, 1893.

**Published:** 1893-05

**Authors:** R. J. Nunn

**Affiliations:** Savannah, Ga.


THE
Southern Medical Record.

A MONTHLY JOURNAL OF MEDICINE AND SURGERY.

VOL. XXIII. ATLANTA, GA., MAY, 1893. NO. 5.

### Original Articles.

### ASPHYXIA NEOPHYTORUM, AN IMPROVED METHOD OF TREATMENT.*

BY R. J. NUNN, M. D., SAVANNAH, GA.

Somewhere about twenty-five or thirty years ago, or perhaps it may be more, Dr. Harvey L. Byrd laid before the medical profession an original method for the resuscitation of the asphyxiated new born.

According to this plan the infant was taken in the hands back downwards and then rythmically doubled upon itself.

This method of Byrd's has been lately resurrected and improved up to the requirements of modern science by Dr. J. Harvie Dew, whose very interesting and elaborate article, "Establishing a new method of artificial respiration in Asphyxia Neonatorum by J. Harvie Dew, M. D., *Medical Record,* March 11th, 1893," could be consulted with advantage.

The method which I am about to describe is one I adopted shortly after Dr. Byrd's was given out. In this method the upper portion of the body is fixed, while the thighs are rythmically pressed against the abdomen thus forcing the diaphragm upward into the thoracic cavity and expelling the air, which rushes in when the legs are extended. The manipulation is about as follows :

* Read before the Medical Association of Georgia, 1893.

The infant being laid upon a table or any other suitable support, the operator stands or sits at either side which happens to be most convenient, he slips the hand which is toward the head of the child palm upward under the back, so as to grasp the ribs and be ready to assist in compressing the chest, and expelling the air, this hand also raises the chest, and permits the head, supported by the edge of the index finger, to fall back the distance desired to make extension and raise the epiglottis. The operator next grasps the legs of the infant with his other hand, back upward near the ankles, the index finger inserted between the legs serves to give a better grip.

Now by steadying the body with one hand, and with the other raising and bending the legs upon themselves, and pressing the thighs upon the abdomen, the diaphragm will be pressed up into the chest and the air expelled therefrom. This operation may be assisted by making pressure upon the ribs with the fingers of the hand supporting the back.

Upon making contrary movement the air again enters the chest.

So far of course there is no change from Byrd's method, the only difference being, that whereas in Byrd's method the upper and lower extremities of the body each swings through an arc of say forty degrees, in this one the lower extremities alone move, and pass through an arc equal to the sum of Byrd's two arcs. Thus it will be observed that in mechanical equivalents both plans are the same.

What advantages then the change?

It enables us to call in the aid of the hot bath, while continuing the artificial respiration, and all without additional help, all that is necessary is to have a tub of suitable length, so that extension can be made, have water of the desired temperature in sufficient quantity to cover the chest of the child, but not the mouth and nose, and then proceed with the manipulation as already described.

This plan of resuscitation has been used by several physicians in this city for years and with good results.

